# Perception of the Sports Social Environment After the Development and Implementation of an Identification Tool for Contagious Risk Situations in Sports During the COVID-19 Pandemic

**DOI:** 10.3389/fspor.2020.610421

**Published:** 2020-11-26

**Authors:** José Ramón Lete-Lasa, Rafael Martín-Acero, Javier Rico-Díaz, Joaquín Gómez-Varela, Dan Río-Rodríguez

**Affiliations:** ^1^Secretaría Xeral para o Deporte, Xunta de Galicia, Santiago de Compostela, Spain; ^2^Grupo de Aprendizaje y Control del Movimiento Humano, Facultade de Ciencias do Deporte e a Educación Física, Universidade da Coruña, A Coruña, Spain; ^3^Facultade de Ciencias da Educación, Universidade de Santiago de Compostela, Santiago de Compostela, Spain; ^4^ATP Entrenamiento Personal, A Coruña, Spain

**Keywords:** COVID-19 virus disease, sport, pandemics, infectious disease transmission, health risk assessments, teaching learning process, risk perception

## Abstract

The present work details the methodological process for creating a tool for the identification of COVID-19 potential contagion situations in sport and physical education before, during, and after practice and competition. It is a tool that implies an educational and methodological process with all the agents of the sports system. This tool identifies the large number of interactions occurring through sports action and everything that surrounds it in training, competition, and its organization. The aim is to prepare contingency protocols based on an exhaustive analysis, risk detection, and proposal of contingency measures trying to reduce the residual risk to a minimum. In a second part, the results of the implementation of this tool in the sports system of Galicia (Spain) are shown revealing the change in perception about the coronavirus transmission in sport of the technicians and the problem for returning to sport for athletes under 18 years.

## Introduction

Pandemic vigilance and reducing the risk of global spreading of the contagious coronavirus (SARS-CoV-2) are now the primary concerns in all sectors. Even more, if we talk about sports, massive events present considerable public health challenges to health authorities, and governments (McCloskey et al., 2020). World Health Organization published a risk assessment tool that enables sports organizers to methodically review critical considerations and risk management steps for hosting an event, assess risks with a weighted-system approach, and factor in risk reduction through various mitigation measures. However, sport is not only preparation for mass events but also daily sports practice. The majority of sports federations do not always count on enough economic resources like mass media sports to use expensive mitigation measures every day (i.e., test, medical staff). Thus, managing and assessing the intrinsic contagion risk inside the practice is of paramount importance.

The everyday sports practitioners are not spectators and have been placed in very harsh circumstances with the current COVID-19 norms, and with specific difficulties in avoiding physical contacts. Unlike shopping in the supermarket, traveling by public transport, or going to school, the sport cannot be done in a static position. In many sports specialties, body movement is mandatory, often intense, sharing, and changing spaces with huge alterations in respiratory frequency. However, both the World Health Organization and the Council of Europe consider the sport a fundamental part of health that should be maintained (Habersaat et al., 2020). Nevertheless, isolation and confinement have limited mobility and increased sedentary behaviors and their harmful effects (Hall et al., 2020). So how do we make it possible to increase risk control in exposure in sports while also resume practice again? The de-escalation of regulations requires sport managers and practitioners to face situations for which they were not prepared, nor have they received guidance with the necessary detail from international or national health and sports authorities. Sports activity implies specific risks, and therefore their identification, analysis, and control must be much more precise and validated than in other sectors. Resume sports practice in compliance with regulations and with the greatest possible benefit for its users, entities, organizations, human and material resources, applying plans and procedures so that the residual risk is minimal is the great goal.

Recognizing the risks and mitigating them will increase the quality of life, providing safeness for the sports community and for each person who belongs to it. The United Nations Office for Disaster Risk Reduction (Schweizer and Renn, 2019; United Nations, 2019) already warned that risk is systemic and, therefore, any study and protocol on risk analysis must integrate the subsystems that comprise it. Institutions and political leaders pursue, through the deployment of risk communication strategies, to reduce the lack of knowledge on specific relevant issues associated with the risk trigger, as well as to reduce and minimize false and distorted information about reality (Mora-Rodríguez and Melero-López, 2021). Communication strategies allow to culturalize the public about why and what the risks are providing expert knowledge on the subject (Rosas-Rodríguez and Barrios-Puga, 2017). Detecting risks is a critical factor in changing social behaviors. Risk analysis is a methodology that helps to understand essential predictors of risk also in sports activities (Asociación Española de Normalización y Certificación (AENOR), 2011, 2018). Risk perception and security are parts of the same continuum, so it is essential to explore in detail the risks to understand how to manage them. Resilience and sustainability are needed to make entities, events, and training viable (Kemp et al., 2020; Stokes et al., 2020).

The sports system of Galicia (Spain) has suffered damage from external causes (crisis, confinement, and de-escalation), and must attend to the peculiarities by specialties so that the situation does not worsen and that it can build new sustainability. So the resilience of a system, community, or society exposed to hazards is to resist, absorb, adapt and recover from the effects of a hazard in a timely and efficient manner, including preservation and restoration of its essential basic structures and functions. Try to resist so that the crisis does not become chronic requires the sports community to be prudent to preserve health, its social balance, and its economic viability. Ideally, authorities should ensure that they fully understand the reality of the situation faced by the people affected by their decisions, drawing on principles of co-production of policy (Han et al., 2020). The sports administration of Galicia and its social environment have promoted a very proactive attitude trusting and supporting each other, seeking to create measures to enable continuity of sport.

Until the development, validation, and implementation of the FISICOVID-DXTGALEGO (FCOVID-DXTG) tool (XUNTA de GALICIA, 2020), the leaders and managers of federations, clubs, town councils, and other entities only had general national legal health regulations and recommendations from non-specific sports organizations like other countries (Pierce et al., 2020). Their plans and protocols have been drawn up with this tool to move prudently toward the practice of individual sports and also fighting and team sports. Thus, this article presents two aims: the development and implementation of a methodological process and a tool for identifying contagions in practice and competition at each time-point, from the transportation for sporting activity, during it, and on the return from it. And the changes in perception of the social environment in each phase of the implementation.

## Methodological Process

This work details the methodological process for creating a tool for the identification of COVID-19 potential contagion situations in sport and physical education before, during, and after practice and competition. It is a tool that implies an educational and methodological process with all the agents of the sports system. This tool identifies the large number of interactions occurring through sports action and everything that surrounds it in training, competition, and its organization. The aim is to prepare contingency protocols based on an exhaustive analysis, risk detection, and proposal of contingency measures trying to reduce the residual risk to a minimum. The creation process, implementation within the sports system of Galicia (Spain), and the operation of this tool is explained in the subsequent subsections. The feedback of this process is shown in the results section. All the processes described took place during lockdown and de-escalation periods between March and the first 2 weeks of April.

### Tool Construction

**Figure 1:**
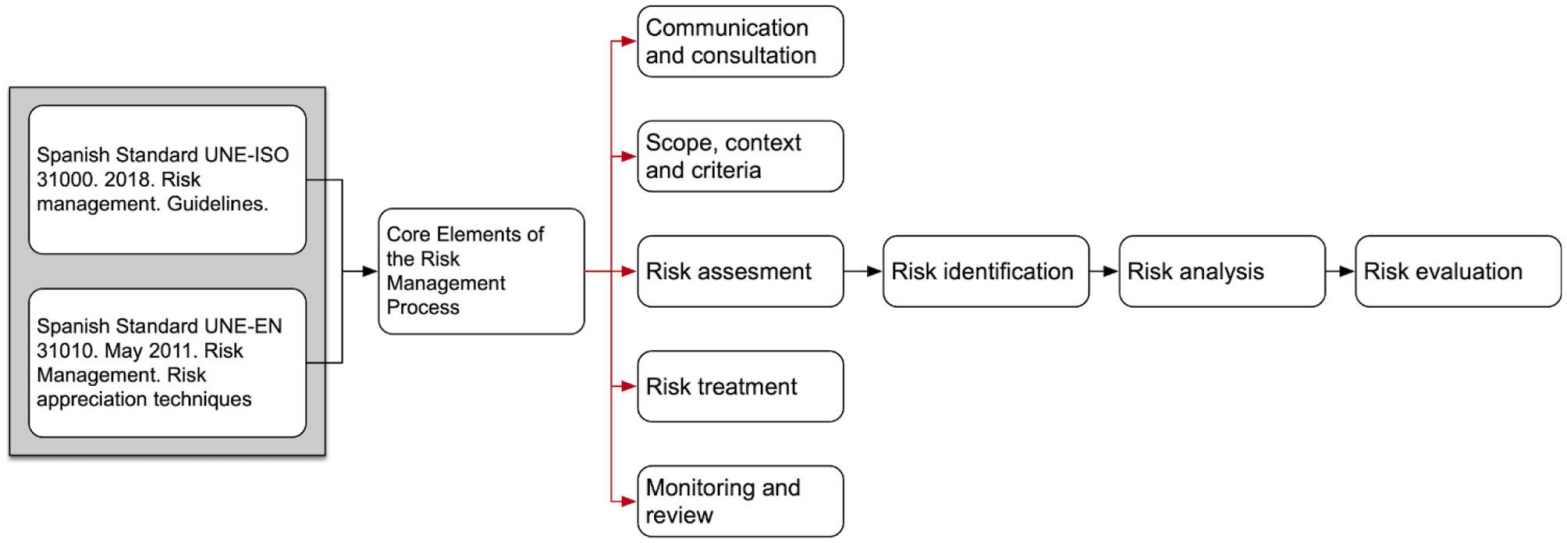
Risk management standards applied for the development of the FISICOVID-DXTGALEGO.

For the development of the tool, the standards UNE-ISO 31000: 2010 and UNE-EN 31010: 2011 (Asociación Española de Normalización y Certificación (AENOR), 2011, 2018) were used as references (Figure 1). It was necessary to determine the variables that would allow the establishment of categorical contextual frameworks for identification requirements of contagion situations during sport. Firstly, 5 experts in health, sports, and risk management in the field of sports were consulted. Two risk assessment techniques were used: a preliminary hazard analysis (PHA), as a simple and inductive method, recognized as highly applicable in novel situations and at the beginning of the development of a project (UNE-EN 31010); and the other the elaboration of verification lists (UNE-EN 31010); both became part of the FCOVID-DXTG tool. Also, meetings for continuous feedback were regularly settled with all agents involved in the process.

#### Levels of Specificity of the Tool

FCOVID-DXTG considers two levels of detail in the assessment of risks. The first level addresses the possible routes of contagion in sports situations at a general level. For its design, the PHA method (Asociación Española de Normalización y Certificación (AENOR), 2011) was used through a systematic and structured process carried out by the administration's group of experts and technicians of the sports administration of Galicia. The objective was to identify dangerous situations through a series of input elements, such as information on the variables and categories involved in the virus transmission processes and, therefore, the potential contagion. The selected variables respond to the characteristics of the risk context, the novelty of the situation, and the limited information available. The objective of developing this level, in this first phase of the design, is to determine an initial list of generic risks for each specialty and applicable as an initial risk matrix. That risk matrix was configured as a checklist to confirm or not the existence of a situation of potential contagion, considering the combination of all the factors involved in the different transmission routes (Table 1) iteratively. The preliminary risk analysis comprises a series of variables and categories:

**Table 1:** Variables of contagion routes for COVID-19 in the FISICOVID-DXTGalego tool.

**Variable**	**Contagion route**
Moment or time condition	Concerning the sporting moment, before practice, during practice, and after practice or competition.
Personal agent involved	Considering the subjects involved in the sporting activity, acting as potential transmitters or infected, and that were categorized according to their functional relationship with the practice environment: *athletes, staff* of the entity or the facility, and *others* not directly related to the practice such as the public, the families that accompany the practitioners.
Condition of virus transmission	*Contact*
	Person to person, directly between agents; indirectly through the use of spaces, sporting tools or surfaces;
	*Aerial*
	Indirect contagion due to the sharing spaces, materials, or surfaces.
	*Ingestion*
	Indirect by sharing food or drinks.

**Figure 2:**
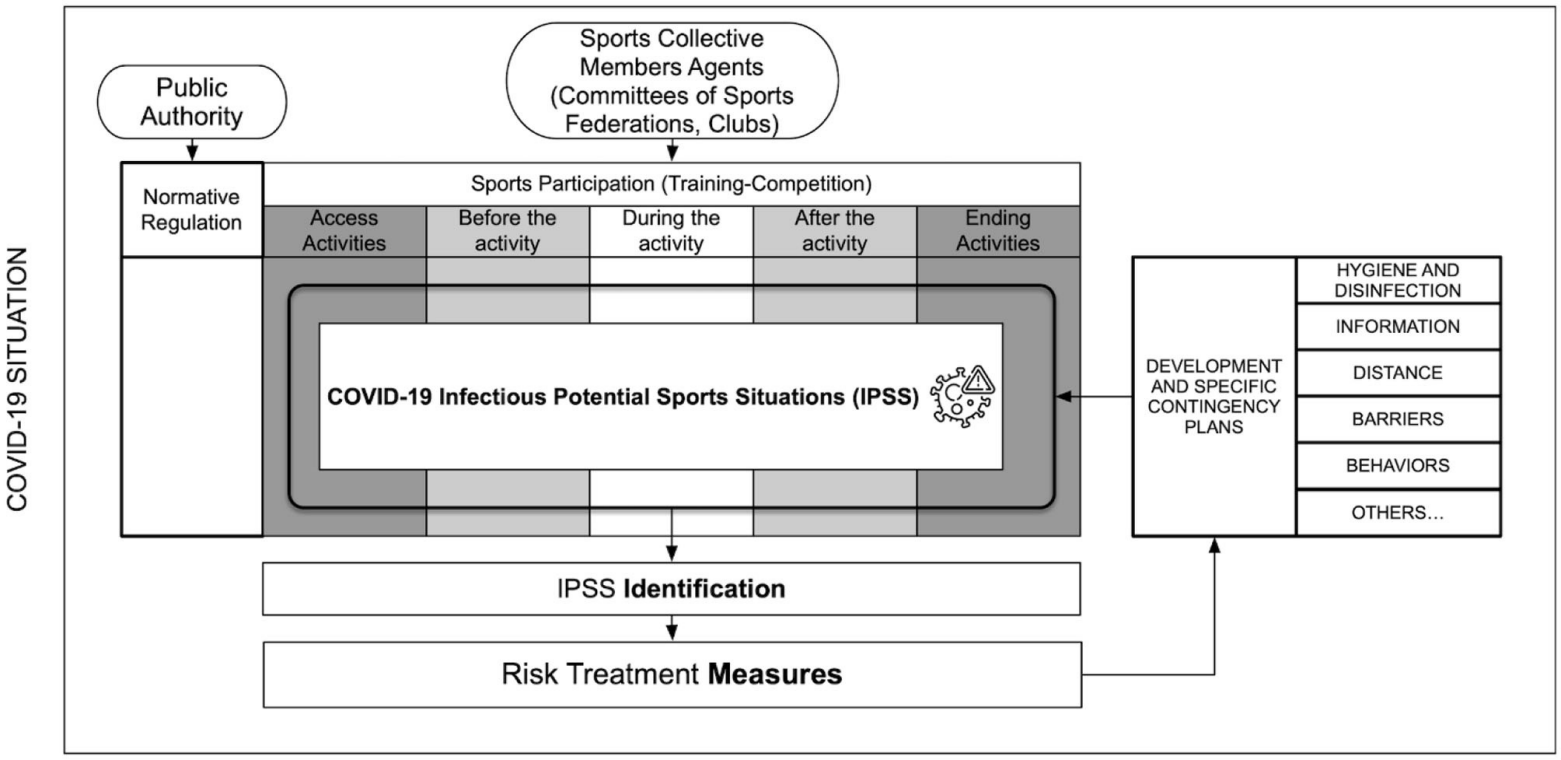
Methodological procedure for applying the FISICOVID-DXTGALEGO.

The second level of detail of the risk matrix describes the specific situations of each sports specialty. Situations are described related to each of the items in the checklist. One or several measures are suggested by the COVID-19 federative officials for the treatment of the risk contagion following the available recommendations and the possibility of adapting sports practice. There was a lack of knowledge about the disease and its transmission during the process, and this changing situation continues even nowadays (Brooks et al. 2020). So, logically the principle of prudence guided this methodological process. Thus, we considered that each situation of potential contagion could entail a risk of transmission and that the different agents involved may be risk agents. Moreover, contemplating the uncertainty generated by the contagion of asymptomatic and pre-symptomatic people, surfaces with fomites, suspension of the virus in aerosols, etc. (Figure 2).

#### Agents

**Figure 3:**
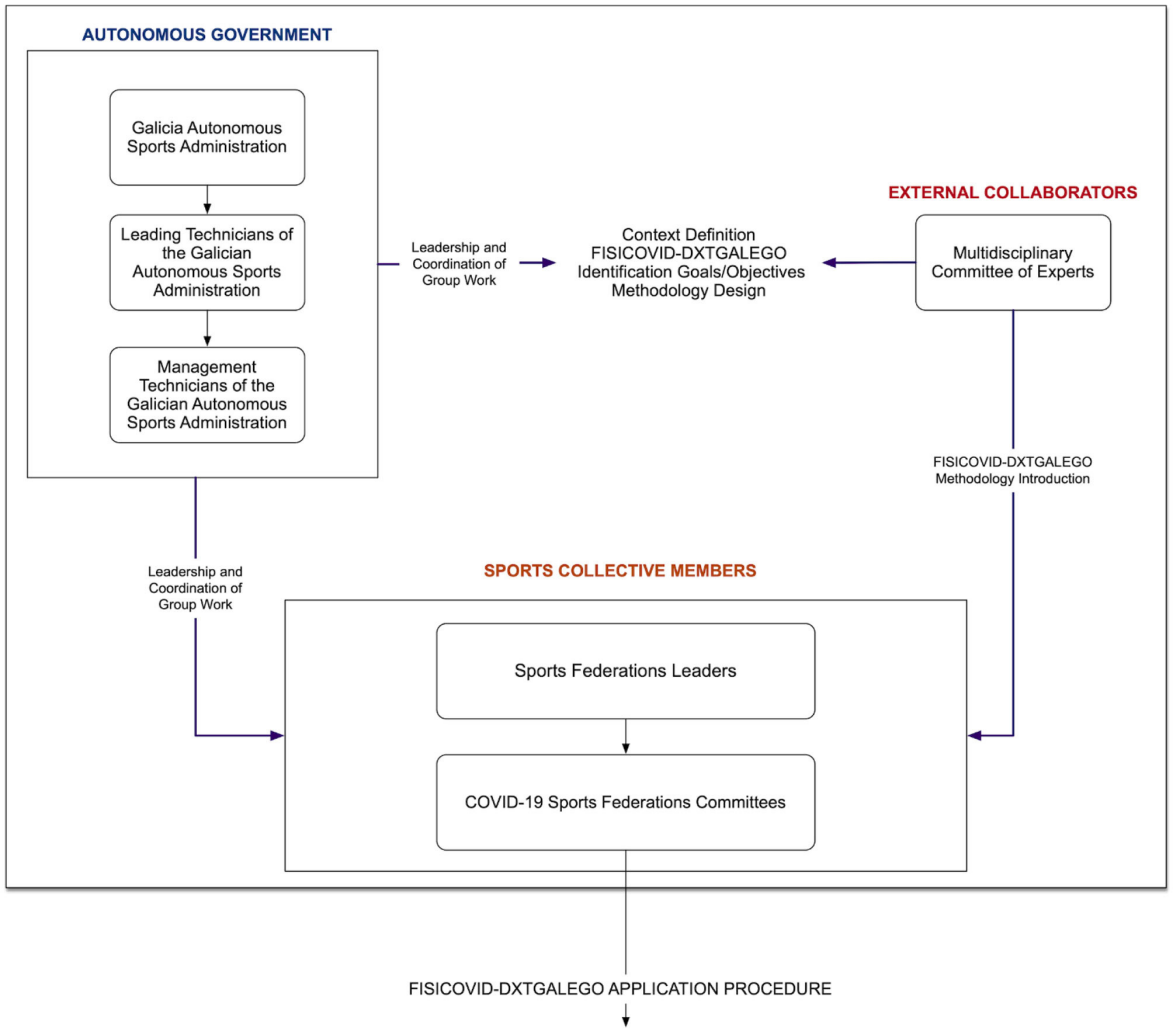
Agents involved in the development of the FISICOVID-DXTGALEGO.

A total of 58 federative officials in charge of the COVID protocols for each federation and its 300 sports specialties participated in this study. The Galician regional sports administration (Secretaria Xeral para o Deporte—Xunta de Galicia), together with a group of experts composed of university specialists in education, sports sciences, and sports management, developed the tool and its methodological process (Figure 3).

### Process of Implementation and Teaching-Learning in Federative Sport

The process to create the FCOVID-DXTG tool implicitly involved the training and learning of federative sports agents during all phases. A first phase of making contact, presenting the problem, and establishing collaboration between the administration and the external multidisciplinary committee of experts; a second phase with the participation of all the federative officials for the delimitation of the context, specification of objectives, and determination of the working method; the third phase for the design, review and testing of the tool and its application procedure with working materials about risk perception in sports; and a final phase including a final product like a web-app, presentation and regulation of use as a tool for reactivating federated sport in Galicia. At the end of the process, each federation developed its protocol based on this FCOVID-DXTG methodological process to achieve the validation of the Autonomous Administration and to be able to resume the practice of their sport.

Permanent communication and consultation between the interested parties involved in this process were part of the risk management process of FCOVID-DXTG. The requirements of the risk assessment were based on the UNE-ISO 31000 and 31010; namely: risk identification, analysis, and evaluation. The treatment of risk through mitigation measures were contemplated in the contingency protocols of each specialty with permanent monitoring, supervision, and actualization with the present scientific information of the virus.

### Operating Mode

**Figure 4:**
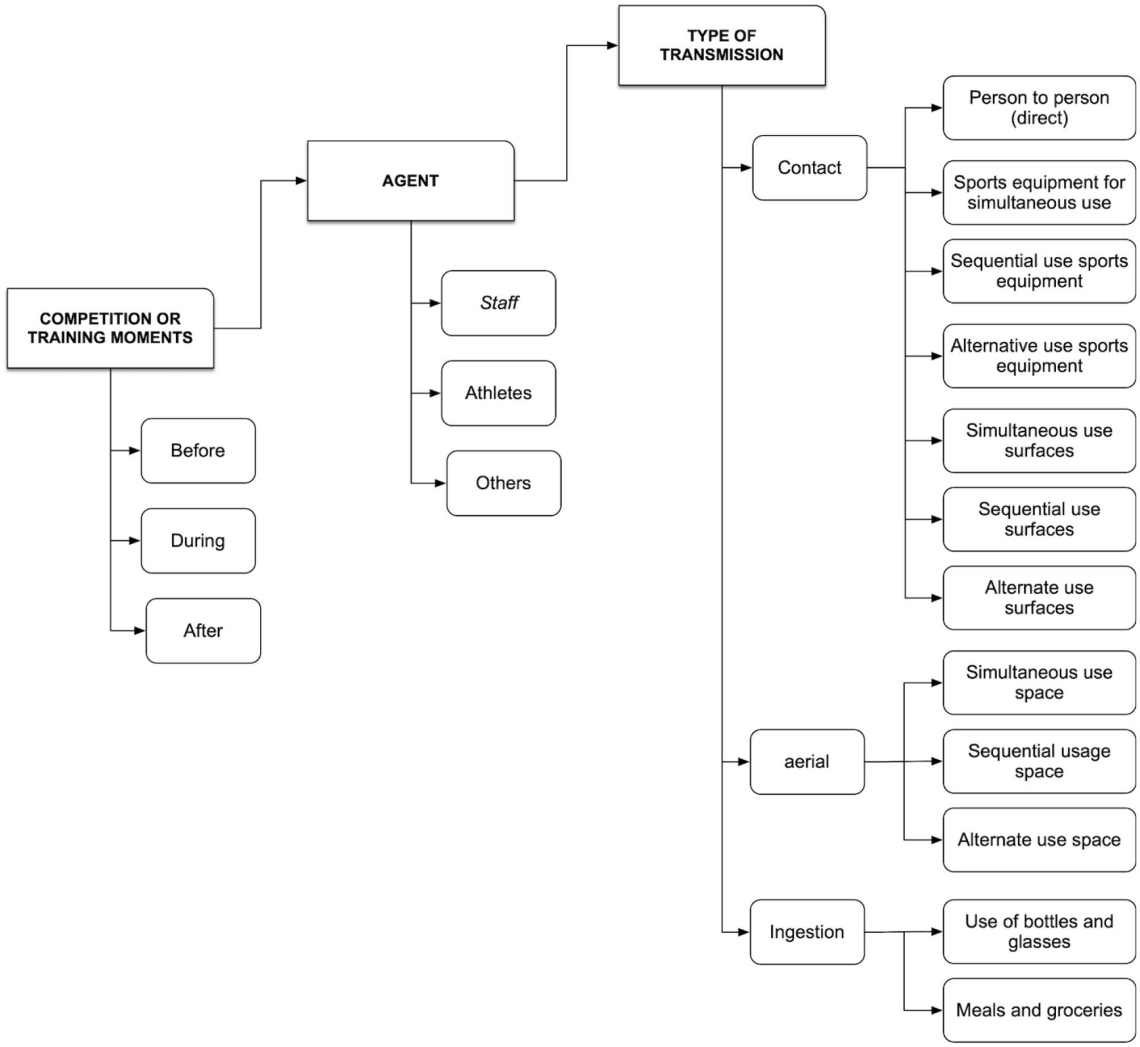
Categories and items for the identification of situations of potential contagion in sport.

This tool was designed to guide the thinking process regarding the risk control of promoters of federated sports activity in training and competitions (Figure 4). At the same time, the organization of the categories of registration/analysis involves a pedagogical process where those responsible evaluate each situation repeatedly, starting from a series of standard items, varying the moments and agents involved according to the specialty. In this way, it is possible to reduce the possibility of forgetting some daily situations, but with the same contagion potential as others more visible and commonly perceived.

The operation of the FCOVID-DXTG can be verified by performing a step by step. First the moment is evaluated (i.e., before training), then all the agents that have action or effect at this time are reviewed (staff, athletes, or others unrelated to sports) and each of the possible routes of contagion (person to person, air, or through food and drink). Within this matrix, all the categories are checked. Once the moment, the agent, the route and the contagion situation have been identified, the FCOVID-DXTG leads us to the first question: Does this situation occur in sports practice? There will only be two possible answers. If the answer is negative, it is necessary to justify why this situation does not occur in sports practice. If the answer is affirmative, then users will have to collect the situation described as precisely as possible in the corresponding section. Once the situation is registered and described in a univocal way, the tool proposes a second question. Can this situation be avoided, or treatment applied to it? Again, the answer is dichotomous: yes/no. In the affirmative case, it is requested to collect one or more measures to counteract this situation. Be it modifications in practice, use of PPE, hygiene and disinfection measures, or others. If the case is negative, there is a situation to be completely avoided or to modify sports practice until the greatest margin of safety can be provided to practitioners.

### Learning Experience on FISICOVID Methodological Process

FISICOVID methodological process must be self-improved in each part of all the processes. It is essential to know how each phase has been developed and what are the points of improvement. Therefore, to achieve this objective, an *ad-hoc* questionnaire was proposed to understand all parts of the process from the design to the implementation of the tool.

### Methods

**Figure 5:**
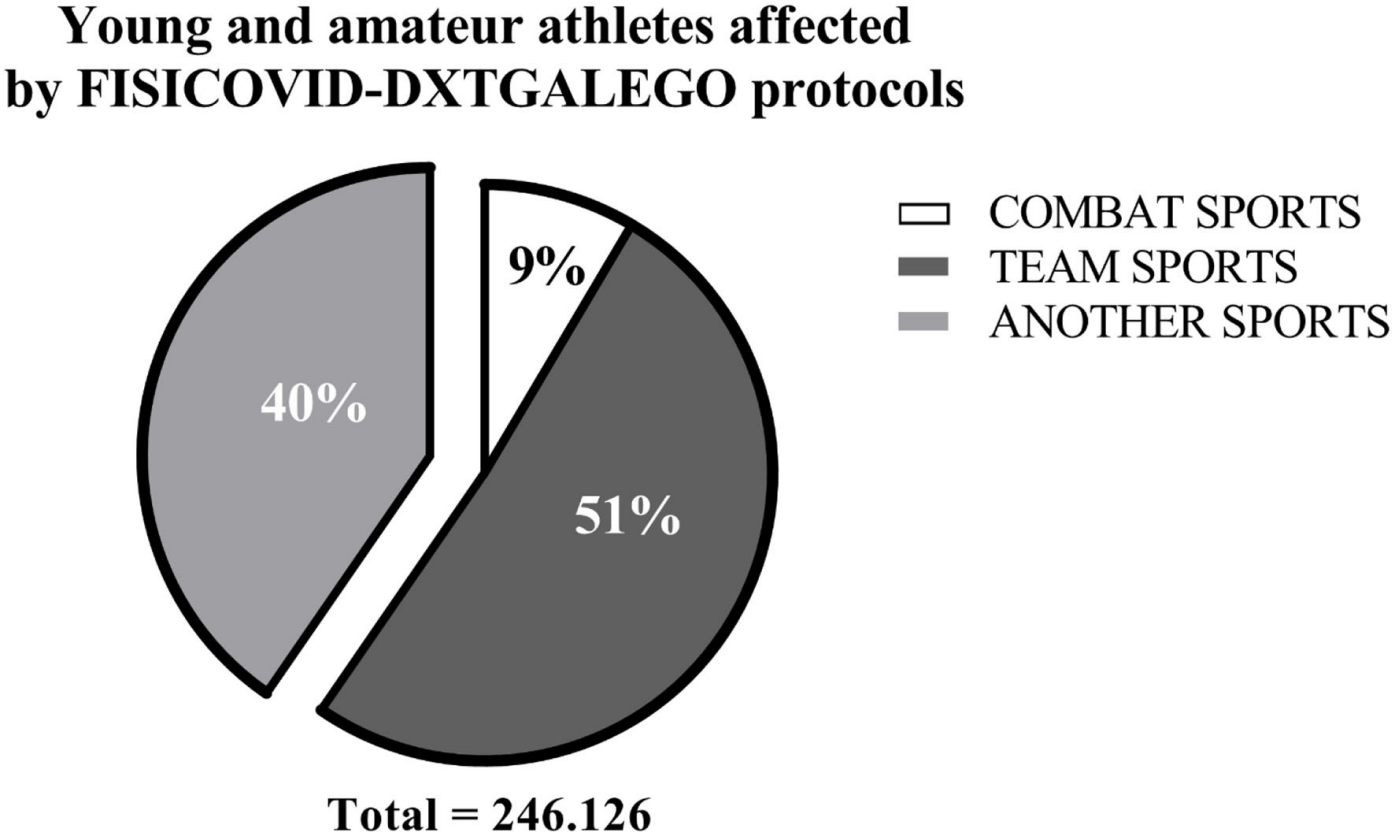
Young and amateur athletes affected by FISICOVID-DXTGalego protocols.

We analyzed how this FCOVID-DXTG methodological process and the working tools were received and implemented by the 58 sports federations with more than 300 specialties, staff, and families with an *ad-hoc* questionnaire in a 1 to 5 Likert scale. Questions explored the implementation process, perceived learning, satisfaction with the tool, perceptions of change for the sports environment, and the expectation for returning to sports after the implementation of the FCOVID-DXTG protocols. Only 9 of the 29 items of the original questionnaire are presented to meet the objectives of this article. The total number of federated athletes affected by protocols based on FCOVID-DXTG was 246.126, distributed as follows: 51% of team sports, and 9% from combat sports. The other 40% was from another sports (Figure 5).

## Results

**Figure 6:**
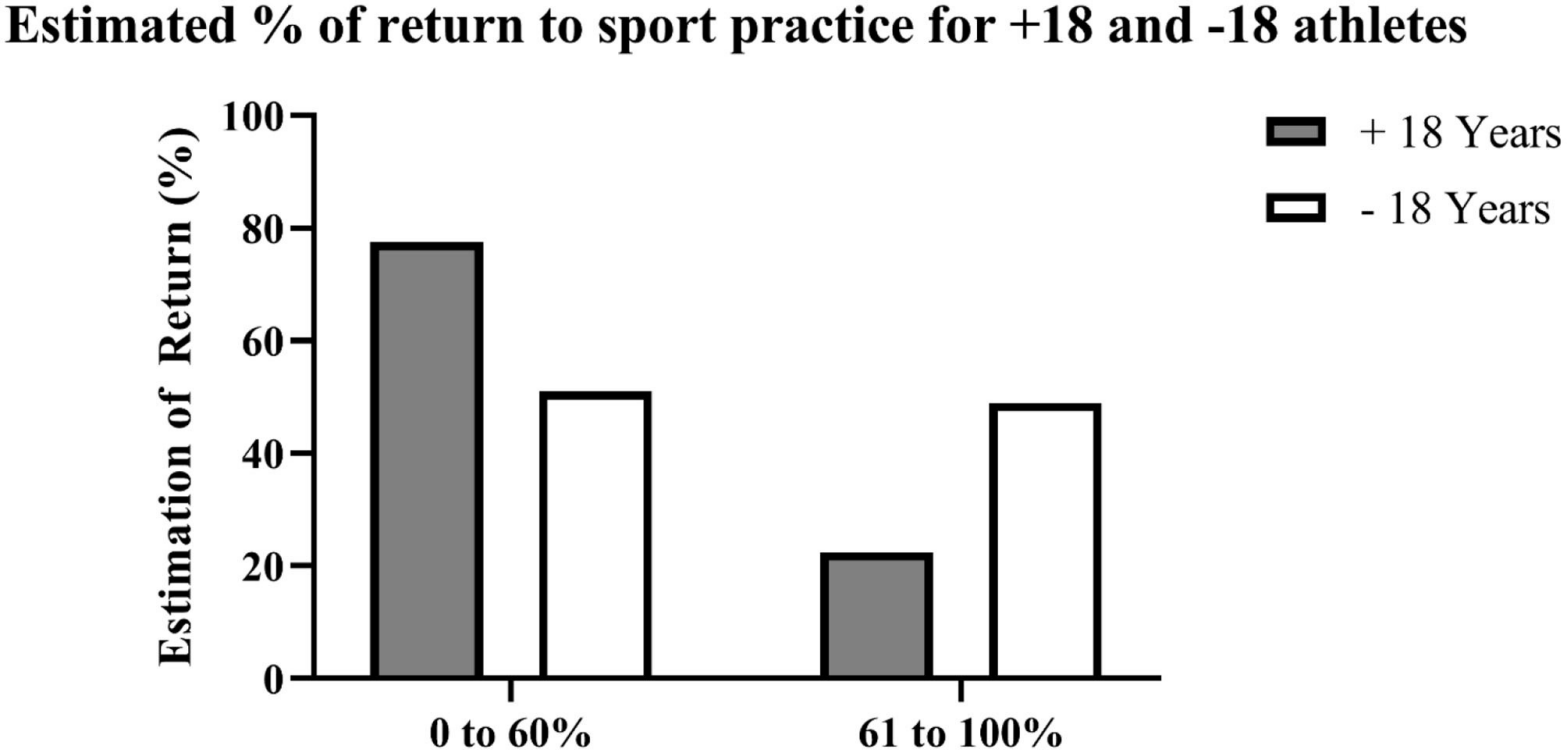
Estimated percentage of return to sport practice for athletes above and under 18 years.

The full results of the questionnaire are displayed in Table 2. Sports officials declared that with the FCOVID-DXTG methodological process they have **learned about identification, assessment, and accuracy in measures to prevent contagion risks in their sport** (4.6 ± 0.6), and they have changed their perception of the infection by coronavirus in their sports (3.9 ± 0.9). They consider the implementation of the protocols improved overall perception about preventing coronavirus infection of the federative collective (4.1 ± 0.9). They declared there was a lack of protocols for avoiding COVID-19 risks before and after sports practice (1.9 ± 1.3) and during the sporting activities (2.6 ± 1.4). They perceived it is easier for young people under 18 to attend sports activities by having protocols with a high level of precision after communicating it among families (4.1 ± 0.9). The satisfaction with the way of working throughout this methodological process was very positive (4.5 ± 0.7). We also asked about their estimation of return to sporting activities for young and adults (Figure 6); 48% declared that returning will be <60% for under 18 years practitioners while 79% answered that more than 60% +18 years practitioners will return. The return estimate is 29 points lower for −18 athletes.

**Table 2:** Selected questions of each item and results related to each part of the teaching-learning process of FISICOVID.

**Question**	**Item**	**Mean**	**SD**
Has your participation in this process led you to learn about identification, assessment, and accuracy in measures to prevent contagion risks in your sport?	Learning in the FISICOVID process	4.6	0.6
Your participation in this process of the elaboration of the protocols FISICOVID-DXTGalego, do you think that it has changed your perception on the infection by coronavirus (COVID-19) in your sport and/or specialties?	Risk perception in the FISICOVID process on your sport	3.9	0.9
With the development and implementation of the protocols, do you think it has improved the overall perception of your federation (leaders, managers, coaches, judges, athletes, families, and other agents) about preventing coronavirus (COVID-19) infection in your sport?	Risk perception in the FISICOVID process of the whole federation	4.1	0.9
Did you have protocols and general measures of NON-SPORTING ENTITIES to avoid risks of contagion before COVID-19 BEFORE and AFTER sports practice?	Before-after protocols of non-sportive entities	1.9	1.3
Did you have protocols and general measures of sports organizations to avoid risks of contagion to covid-19 during sports?	During Sport Protocols of sportive entities	2.6	1.4
Do you think that having a precise and specific protocol and communicating it among families has a positive influence on the decision for children and young people UNDER 18 to attend sports activities?	Protocol influence for returning to activity of children under 18	4.1	0.9
What is your level of satisfaction with the FORM OF WORK CARRIED OUT (collectively, openly and sharing information) in the development of the whole FISICOVID-DXTGalego process?	SATISFACTION with work Methodology	4.5	0.7
What is your estimate for the return to sport of those under 18 years of age?	Estimation of Return under 18 years	(In text)	
What is your estimate for the return to sport of those over 18 years of age?	Estimation of Return above 18 years	(In text)	

## Discussion

The participation of federative sports promoters in the FCOVID-DXTG methodological process has changed its safety perception after its implementation. The families have been provided with detailed information with the protocols coming up from this tool, making more accessible the return to sport for children. However, 30 points of difference between estimations for under and +18 practitioners stress the importance of an existing barrier for children and young people to resume their sports habits after a crisis like COVID-19 pandemics.

### Methodological Process

The main objective has been to generate a methodological procedure to search for safe, specific, and flexible solutions that allow the identification of situations of potential contagion in the highly complex field of sports participation. At the end of the application of the FCOVID-DXTG methodological process, each organization obtains information documenting a complete list of risks for each sports specialty, with the applicable measures to minimize the residual risk to be included in its protocol for the coronavirus. The tool establishes a homogeneous language for communication and feedback between sports specialties and entities, being a service to the sporting community that could not solve in isolation, due to its limited management capacity and resources. The union of administration, federations, and universities with FCOVID-DXTG represent an example of an open way of working as international authors suggested (Habersaat et al., 2020) and (Kemp et al., 2020). Also, The Council of Europe (06/26/2020) urged the governments of the member states to “*support the recovery and promote the sustainable development of the sport, to provide support to the sports sector through programs and EU funds. To study the possibilities within the framework of relevant horizontal measures and initiatives of EU initiatives for recovery. To promote the continuity of regular sport funding programs and initiatives, especially for grassroots sports organizations*.” Galician sports administration had anticipated in March to what today are the recommendations of the Council of Europe assuming the greatest possible implication for protection and support of the sports sector: Promoting broad cooperation and collaborating at all levels to resume sports activities safely, gradually and cautiously, evaluating all the potential risks.

The development and application of FCOVID-DXTG tackle the complexity of the federated sports organization before and after the practice (McCloskey et al., 2020; Wordl Health Organization, 2020). It also provides a solution against generic and non-specific protocols that did not promote the identification of contagion risks during the own motor activity of each sports specialty without leaving loopholes for the appearance of unexpected situations where contagion can occur and facilitating its tracking and monitoring. Besides, FCOVID-DXTG can be adapted to other areas of action, such as the education sector or private gyms. To the author's knowledge, this tool is being applied in the field recreative and educational summer camps, agreeing in many of the evaluated items with the document that the Centers for Disease Control and Prevention (CDC) of USA have published as a checklist for camping activities (Suggestions for Youth Programs and Camps: Readiness and Planning Tool) (U.S. Centers for Disease Control Prevention, 2020).

### Perception of the Sports Social Environment

Change in perception of risk is the first step to manage it with guarantees. In the FCOVID-DXTG methodological process, the federative officials, responsible for the sports organization, declared that their ability to identify contagion risk was highly improved as well as their virus perception. This is of great importance since Spain has been one of the countries most affected by the pandemic, and the level of information and counter-information has been enormous since having a well-trained criterion would make fewer mistakes (Mora-Rodríguez and Melero-López, 2021).

Participation in all parts of the process generated great satisfaction perceived by the federative leaders, who declared a very high satisfaction for making better protocols throughout FCOVID-DXTG. This is in agreement with the recommendations of Schweizer and Renn (2019) on the risk mitigation with systemic hazards like COVID-19 pandemics. Also, FCOVID-DXTG methodological process has met the criteria of *Crisis and Emergency Risk Communication* working model of Reynolds and Seeger (2007), promoting changes in the behavior of sports agents to reduce the likelihood of harms; developing consensual recommendations by experts and first responders; trying to reduce uncertainty improving self-efficacy; generating empathy, reassurance, and reduction in emotional turmoil and creating better public understanding of new risks and new understandings of risk as well as new risk avoidance behaviors and response procedures.

The estimation of federative officials for the return to sporting activities for young will be <60%; this is a significant problem since the resumption of sport and recreation activities can contribute to physical, psychological, andemotional benefits to societies emerging from the COVID-19 restrictions (Hughes, 2020). And this not only affects federated sports but all sporting promotion activities such as the Galician sports program “XOGADE” where 128.614 children practice sports every week during school hours are also affected (Rico-Díaz et al., 2020). However, they believe that it is easier for children to return to sport by having protocols with a high level of precision by its influence on the decision of families respect to non-sport-specific general protocols overcoming the deleterious psychological effects of a lockdown (Brooks et al., 2020). The Sports Innovation Institute at IUPUI, a partnership between Indiana and Purdue universities in Indianapolis, and Grand Park Sports Campus (Westfield, Ind.) collaborated to better understand how COVID-related adaptations are perceived by parents, athletes, coaches, officials, and administrators with good results in how specific adaptions are received by these stakeholders who are looking to return to youth sports in a timely, but safe manner with a positive response about mitigation measures like sanitizing playing areas, mask use, monitoring of social-distancing guidelines and limiting personal contact between players (Pierce et al., 2020).

Some proposals and recommendations on this topic were published. However, the critical point of sports interactions was not stressed enough. Youth Sports Program FAQs from the US government agency CDC, through its National Center for Immunization and Respiratory Diseases (NCIRD) (U.S. Centers for Disease Control Prevention, 2020) strongly recommended using the facemask to solve the sports interaction issue. Others, like the proposal for the active tourism of Aragon (Turismo Deportivo Aragón, 2020), also developed a checklist system for the control of risks with an external consultant. The closest proposal to control over the real game has been made by Phillips et al. (2020) authors with a Team Sport Risk Exposure framework to provide stakeholders with a practical method to inform discussions around the return-to-sport. Thus, to the best of the author's knowledge, this is the first tool that had addressed the reality of the complexity, diversity, and quantity of situations that sports practice implies before, during, and after training and competition.

## Conclusions

Sharing information and having a common language for communication and feedback have been provided new solutions for the technicians to the common and specific problems of the sports specialties. Returning to sport with a safe practice the technicians consider that a safe return to sport is possible with an in-depth analysis of the risks and contacts of each sport specialty. The problem to be addressed is framed in the interaction of two determining variables of the situation, the possibility of contagion and that sport involves a whole series of situations and behaviors that require greater precision in the measures for its prevention. Given this, it is worth asking: Are the general measures to prevent contagion sufficient and applicable for sports practices? How can training and sports competition be resumed with guarantees? And in all sports specialties, the contagion prevention measures to be adopted would be the same? Facing with a changing situation and uncertainty, we need tools that make it possible to configure contingency protocols, so that, in each specialty, once preventive measures are applied, residual risk is reduced to a minimum.

## Data Availability Statement

The raw data supporting the conclusions of this article will be made available by the authors, without undue reservation.

## Ethics Statement

The Galician Sports Administration (Spain) approved all the procedures. The participants provided their written, informed consent to participate in this study.

## Author Contributions

JL-L did manage the sports and political administration to work together. JR-D coordinated all the working groups. RM-A created the intervention model. JR-D, RM-A, JG-V, and DR-R designed the tool. RM-A, JG-V, and DR-R contributed equally to the development of the manuscript. All authors approved the manuscript in its final form.

## Conflict of Interest

The authors declare that the research was conducted in the absence of any commercial or financial relationships that could be construed as a potential conflict of interest.

## References

[B1] Asociación Española de Normalización y Certificación (AENOR) (2011). Gestión Del Riesgo. Técnicas de Apreciación Del Riesgo (UNE-EN 31010). Available online at: https://www.aenor.com/normas-y-libros/buscador-de-normas/une/?c=N0047287

[B2] Asociación Española de Normalización y Certificación (AENOR) (2018). UNE-ISO 31000:2018. Available online at: https://www.aenor.com/normas-y-libros/buscador-de-normas/une?c=N0059900

[B3] Brooks S. K. Webster R. K. Smith L. E. Woodland L. Wessely S. Greenberg N.. (2020). Rapid review the psychological impact of quarantine and how to reduce it : rapid review of the evidence. Lancet 395, 912–920. 10.1016/S0140-6736(20)30460-8 32112714 PMC7158942

[B4] Habersaat K. B. Betsch C. Danchin M. Sunstein C. R. Böhm R. Falk A.. (2020). Ten considerations for effectively managing the COVID-19 transition. Nat. Hum. Behav. 4, 677–687. 10.1038/s41562-020-0906-x 32581299

[B5] Hall G. Laddu D. R. Phillips S. A. Lavie C. J. Arena R. (2020). A tale of two pandemics: how will COVID-19 and global trends in physical inactivity and sedentary behavior affect one another? Prog. Cardiovasc. Dis. 10.1016/j.pcad.2020.04.005. [Epub ahead of print]. 32277997 PMC7194897

[B6] Han E. Mei M. Tan J. Turk E. Sridhar D. Leung G. M.. (2020). Lessons Learnt from Easing COVID-19 Restrictions : an Analysis of Countries and Regions in Asia Pacific and Europe. Health Policy 6736, 1–10. 10.1016/S0140-6736(20)32007-9 32979936 PMC7515628

[B7] Hughes D. (2020). In the frame, road map for Australian sport on an uncertain journey through COVID-19. J. Sci. Med. Sport 23, 636–638. 10.1016/j.jsams.2020.05.003 32416971 PMC7198417

[B8] Kemp S. Cowie C. M. Gillett M. Higgins R. Hill J. Iqbal Z.. (2020). Sports medicine leaders working with government and public health to plan a ‘return-to-sport’ during the COVID-19 pandemic: the UK's collaborative five-stage model for elite sport. Br. J. Sports Med. 10.1136/bjsports-2020-102834. [Epub ahead of print]. 32661129

[B9] McCloskey B. Zumla A. Ippolito G. Blumberg L. Arbon P. Cicero A.. (2020). Mass gathering events and reducing further global spread of COVID-19: a political and public health dilemma. Lancet 395, 1096–1099. 10.1016/S0140-6736(20)30681-4 32203693 PMC7138150

[B10] Mora-Rodríguez A. Melero-López I. (2021). News consumption and risk perception of Covid-19 in Spain. [Seguimiento informativo y percepción del riesgo ante la Covid-19 en España]. Comunicar. 66 10.3916/C66-2021-06

[B11] Phillips G. Kemp S. P. T. Griffin S. A. Readhead C. Pearce N. Stokes K. A. (2020). A team sport risk exposure framework to support the return to sport. Br. J. Sports Med. Available online at: https://blogs.bmj.com/bjsm/2020/07/01/a-team-sport-risk-exposure-framework-to-support-the-return-to-sport/10.1136/bjsports-2021-104225 34187786

[B12] Pierce D. Stas J. Feller K. Knox W. (2020). Preparing sports venues and events for the return of youth sports COVID-19 : return to youth sports. Sports Innovation J. 1, 62–80. 10.18060/24144

[B13] Reynolds B. Seeger M. W. (2007). Crisis and emergency risk communication as an integrative model crisis and emergency risk communication as an integrative model. J. Health Commun. 10, 43–55. 10.1080/10810730590904571 15764443

[B14] Rico-Díaz J. Navarro-Patón R. Benavides-Alonso D. (2020). XOGADE: programa de deporte en edad escolar de la xunta de galicia. El papel de las agrupaciones deportivas escolares. J. Sport Health Res. 12(Supl. 2), 137–144.

[B15] Rosas-Rodríguez M. E. Barrios-Puga A. (2017). Comunicación de riesgo, cambio climático y crisis ambientales. Chasqui. Rev. Latinoamericana Comun. 136, 179–194. 10.16921/chasqui.v0i136.3294

[B16] Schweizer P-J. Renn O. (2019). Governance of systemic risks for disaster prevention and mitigation. Disaster Prev. Manag. 28, 862–874. 10.1108/DPM-09-2019-0282

[B17] Stokes K. A. Jones B. Bennett M. Close G. L. Gill N. Hull J. H.. (2020). Returning to play after prolonged training restrictions in professional collision sports. Int. J. Sports Med. 10.1055/a-1180-3692. [Epub ahead of print]. 32483768 PMC7799169

[B18] Turismo Deportivo Aragón (2020). Guía de Autoformación Recomendaciones Para El Desarrollo Del Plan de Contingencia Por El Coronavirus. Available online at: https://www.dropbox.com/s/cictlb7wqbqfr7x/TDA%20-%20AKTIBA%20Protocolo%20medidas%20COVID19.xlsx?dl=0

[B19] U.S. Centers for Disease Control and Prevention (2020). Suggestions for Youth Programs and Camps: Readiness and Planning Tool CDC Readiness and Planning Tool to Prevent the Spread of COVID-19 Among Campers. (accessed August 01, 2020).

[B20] United Nations (2019). The ripple effect of covid-19. Global assessment report. Available online at: https://www.undrr.org/drr-and-covid-19

[B21] Wordl Health Organization (2020). Considerations for Sports Federations/Sports Event Organizers When Planning Mass Gatherings in the Context of COVID-19. Available online at: https://apps.who.int/iris/bitstream/handle/10665/331764/WHO-2019-nCoV-Mass_Gatherings_Sports-2020.1-eng.pdf

[B22] XUNTA de GALICIA (2020). FISICOVID - DXTGALEGO Ferramenta de identificación de situacións de contaxio no deporte galego. Available online at: https://fisicovid.dxtgalego.gal/

